# Multivariate risk assessment and analysis of brain injury in high-risk infants in neonatal intensive care unit

**DOI:** 10.3389/fped.2026.1831483

**Published:** 2026-05-21

**Authors:** Daodan Huang, Rong Zhou, Yikang He, Yao Sun, Yongfang He, Min Zhu

**Affiliations:** 1 Department of Pediatrics, The Second People’s Hospital of Wuhu, Wuhu, Anhui, China; 2 Department of Rehabilitation, Children's Hospital of Nanjing Medical University, Nanjing, Jiangsu, China

**Keywords:** brain injury, high-risk infant, neonatal intensive care unit, prediction model, risk factor

## Abstract

**Background:**

To explore risk factors, establish, and validate a risk prediction model for brain injury in high-risk infants in neonatal intensive care units (NICU).

**Methods:**

A case-control study was conducted involving 819 high-risk infants admitted to the Second People's Hospital of Wuhu City from March 2019 to March 2023. Participants were divided into a modeling group (*n* = 655) and a validation group (*n* = 164) at an 8:2 ratio. In the modeling cohort, brain injury was diagnosed by cranial ultrasound or magnetic resonance imaging (MRI). Intraventricular hemorrhage (IVH) was graded using the Papile classification and Volpe classification, and hypoxic-ischemic encephalopathy (HIE) was staged according to the Sarnat staging system. The modeling group was further divided into a brain injury group (*n* = 131) and a non-brain injury group (*n* = 524) based on the presence or absence of brain injury. Clinical data, maternal pregnancy status, and laboratory indicators were monitored and recorded. Risk factors for brain injury were analyzed through univariate and logistic regression analyses. Independent risk factors identified in the modeling group were used to establish a predictive model. A nomogram was developed to visualize the prediction model using R-4.0.2 software. The model's performance was evaluated and validated using discrimination and calibration assessments in both groups.

**Results:**

Meconium-stained amniotic fluid (MSAF), neonatal respiratory distress syndrome (NRDS), and pregnancy-associated anemia were independent risk factors for brain injury (*P* < 0.05). The 5-minute Apgar score served as a protective factor against brain injury (*P* = 0.01). Additionally, low umbilical artery PH and low base excess (BE) were associated with a higher brain injury (*P* < 0.05). Furthermore, higher serum levels of brain-derived neurotrophic factor (BDNF) and 25(OH)D were protective factors (*P* < 0.05). The predictive model showed an area under the ROC curve (AUC) of 0.935, indicating high accuracy.

**Conclusion:**

Meconium-stained amniotic fluid (MSAF), NRDS, pregnancy-associated anemia, and low umbilical artery PH, and low BE are independent risk factors for brain injury. A higher 5-minute Apgar score and elevated serum levels of BDNF and 25(OH)D are protective factors. The risk prediction model established on the basis of Papile, Volpe, and Sarnat standardized criteria shows favorable performance and can be used for early risk stratification of brain injury in high-risk NICU infants.

## Introduction

A neonatal intensive care unit (NICU) is a specialized setting designed for the continuous monitoring, prompt diagnosis, and effective management of high-risk newborns (1). Among conditions encountered in NICUs, brain injury in high-risk infants is particularly common. This condition is defined as non-progressive cerebral damage occurring between the antenatal period and the first postnatal month (2, 3), caused by various intrauterine or extrauterine insults. The pathogenesis of brain injury in high-risk neonates is complex, and its clinical manifestations are often subtle. Without timely intervention and effective treatment, permanent neurological sequelae (4, 5) may develop, including delayed brain development, intellectual disability, and epilepsy. Accumulating evidence indicates that hypoxia–ischemia and systemic inflammation are the primary contributors to neonatal brain injury (6, 7). To date, considerable research has been conducted to identify risk factors for neonatal brain damage, with most studies focusing on preterm infants (8, 9), especially those born extremely or very preterm. Compared with term infants, preterm neonates exhibit immature anatomical and physiological development. Consequently, their brain tissue is more susceptible to softening, edema, hemorrhage, or necrosis under perinatal insults such as birth trauma or hypoxia–ischemia (10, 11). Epidemiological data indicate that brain injury accounts for approximately 15% of neonatal disorders and represents one of the major complications among preterm infants (12). Apart from preterm infants, NICUs routinely manage conditions such as NRDS, congenital heart disease, hemolytic disease, and various genetic disorders. However, few studies have conducted a comprehensive, multifactorial risk assessment of brain injury in this broader high-risk NICU population.

In this hospital-based case-control study, 819 high-risk neonates admitted to the NICU of the Second People's Hospital of Wuhu between March 2019 and March 2023 were enrolled. Using simple random sampling, the cohort was divided at an 8:2 ratio into a derivation cohort (*n* = 655) and a validation cohort (*n* = 164). Clinical data were extracted from the electronic medical record system. Neonatal characteristics, maternal obstetric history, and laboratory indices were recorded for both groups. Univariate analysis and multivariable logistic regression were conducted to identify factors associated with brain injury in high-risk NICU neonates. Subsequently, a predictive risk model was constructed and visualized as a nomogram using R-4.0.2 software. The model's performance was evaluated and internally validated. This study systematically identifies risk factors for brain injury among high-risk NICU infants treated at the Second People's Hospital of Wuhu. It provides an evidence-based tool for the early prevention, detection, and treatment of neonatal brain injury.

## Materials and methods

### General information

Medical records of 819 high-risk neonates admitted to the NICU between March 2019 and March 2023 were retrospectively reviewed. Neonates were allocated at an 8:2 ratio into a derivation cohort (*n* = 655) and an internal validation cohort (*n* = 164). For model development, the derivation cohort was further divided into a brain injury group (*n* = 131) and a no brain injury group (*n* = 524) based on documented brain injury.

Diagnostic criteria for brain injury:

1. Intraventricular hemorrhage: Diagnosed by cranial ultrasound and graded by the Papile classification and Volpe classification; grade ≥ II was defined as brain injury (13, 14).
2. Hypoxic-ischemic encephalopathy: Diagnosed according to the Sarnat clinical staging system, confirmed by cranial ultrasound or MRI showing cerebral edema, white matter injury, or infarction (15, 16).
3. Congenital genetic brain injury, central nervous system malformations, and chromosomal abnormalities were excluded (17).

Inclusion Criteria: (1) Neonates admitted to the hospital within 24 h after birth and within the neonatal period (0–28 days). (2) Mothers who completed regular and standardized prenatal check-ups at our hospital, with complete neonatal examination data post-birth. (3) Obtained informed consent from the neonate's legal guardian and ethical approval from our institutional ethics committee, complying fully with medical ethics requirements.

Exclusion Criteria: (1) High-risk infants admitted more than 24 h after birth for observation and treatment. (2) Infants with other concurrent diseases, including congenital heart disease, digestive tract malformations, malignant tumors, congenital malformations, congenital hereditary brain injury, or hereditary metabolic diseases. (3) Cases involving treatment abandonment by family, infant death during hospitalization, or incomplete clinical data due to transfer or interrupted treatment.

### Research methods

Blood gas indices (pH, PaO_2_ [mmHg], PaCO_2_ [mmHg], and BE [mmol/L]) were measured within 30 min after sampling using a Siemens RAPIDPoint 500 analyzer. Full blood counts (RBC [×10^12^/L], WBC [×10^9^/L], Hb [g/L], and PLT [×10^9^/L]) were obtained from 50 µL of arterial blood using a CAL8000 automated hematology analyzer. Liver function tests were conducted on an AU-5800 chemistry analyzer. Serum brain-derived neurotrophic factor (BDNF) was measured using a commercial enzyme-linked immunosorbent assay (ELISA) kit (Cat. No.: E-EL-H0020, Elabscience Biotechnology Co., Ltd., Wuhan, China) according to the manufacturer's instructions. For 25-(OH)D analysis, 2 mL venous blood was collected upon admission, centrifuged at 3,000 rpm (radius: 8 cm, 10 min), and serum was stored at −80 °C until analyzed by a Cobas e601 electrochemiluminescence immunoassay analyzer (Roche, Switzerland).

### Statistical analysis

Statistical analyses were performed using SPSS version 22.0. Categorical variables (sex, gestational age, parity, and clinical characteristics) are presented as frequencies and compared by *χ*^2^ tests. Continuous variables were tested for normality; normally distributed data (birth weight, umbilical-cord blood gases, routine blood counts) are expressed as mean ± standard deviation (SD) and compared using independent-sample *t*-tests. Non-normally distributed data (5-min Apgar score, WBC count) are presented as median (interquartile range) and compared by Mann–Whitney *U* tests (*Z* statistic). Predictors were selected by bidirectional stepwise selection and incorporated into a logistic regression model (rms package, lrm function, penalty = 0.1). Model performance was internally validated through 500 bootstrap calibration curves (goodness-of-fit) and receiver operating characteristic (ROC) analysis (discrimination; AUC) (18, 19). The final model was visualized as a nomogram to quantify the contribution of each clinical variable to individual risk. Statistical significance was defined as *P* < 0.05 (two-tailed *α* = 0.05).

## Results

### Baseline characteristics

Gestational age, sex, parity, and birth weight showed no significant differences between the brain-injury and no-brain-injury groups (all *P* > 0.05, Table 1).

**Table 1:** Comparison of demographic data between brain-injury and non-brain injury groups in high-risk neonates.

Variables	Classification	Brain injury group (*n* = 131)	Non-brain injury group (*n* = 524)	*χ*^2^/*t*	*P*
Gender	Male	76 (58.0)	283 (54.0)	0.68	0.41
	Female	55 (42.0)	241 (46.0)		
Gestational age	Preterm	36 (27.5)	107 (20.5)	3.06	0.08
	Term	95 (72.5)	417 (79.5)		
Parity	≤2	100 (76.3)	431 (82.3)	2.39	0.12
	≥3	31 (23.7)	93 (17.7)		
Birth Weight		2,652.19 ± 607.78	2,742.77 ± 587.69	1.56	0.11

### Antenatal clinical data

Placental abnormalities, meconium-stained amniotic fluid (MSAF), and intrauterine distress occurred significantly more frequently in the brain-injury group compared to the no-brain-injury group (all *P* < 0.05). However, premature rupture of membranes and intrauterine infection did not differ significantly between groups (both *P* > 0.05, Table 2).

**Table 2:** Comparison of antenatal clinical data between brain injury and non-brain injury groups in high-risk neonates.

Variables		Brain injury group (*n* = 131)	Non-brain injury group (*n* = 524)	*χ* ^2^	*P*
Premature rupture of membranes (PROM)	Yes	30 (22.9)	104 (19.8)	0.61	0.43
	No	101 (77.1)	420 (80.2)		
Placental abnormalities	Yes	7 (5.3)	46 (18.8)	1.67	0.19
	No	124 (94.7)	478 (91.2)		
Umbilical cord abnormalities	Yes	54 (41.2)	159 (30.3)	5.65	0.02*
	No	77 (58.8)	365 (69.7)		
MSAF	Yes	34 (26.0)	91 (17.4)	5.01	0.03*
	No	97 (74.0)	433 (82.6)		
Intrauterine distress	Yes	40 (30.5)	98 (18.7)	8.23	0.01*
	No	91 (69.5)	426 (81.3)		
Intrauterine infection	Yes	9 (6.9)	19 (3.6)	2.69	0.11
	No	122 (93.1)	505 (96.4)		

* Statistical significance (*P* < 0.05).

### Neonatal clinical data

Neonatal clinical data differed significantly between high-risk infants who developed brain injury and those who did not. The median 5-minute Apgar score was 8 (IQR: 8–10) in the brain-injury group compared to 9 (IQR: 8–10) in the no-brain-injury group (*Z* = 3.21, *P* = 0.01). Furthermore, both neonatal pulmonary infection and RDS were significantly more common in the brain-injury group (both *P* < 0.05, Table 3).

**Table 3:** Comparison of neonatal clinical data between brain injury and non-brain injury groups in high-risk neonates.

Variables		Brain injury group (*n* = 131)	Non-brain injury group (*n* = 524)	*χ*^2^/*Z*	*P*
5-Minute Apgar score		8 (8, 10)	9 (8, 10)	3.21	0.01*
Congenital malformations	Yes	2 (1.5)	6 (1.1)	0.13	0.72
	No	129 (98.5)	518 (98.9)		
Pulmonary infection	Yes	62 (47.3)	196 (37.4)	4.32	0.04*
	No	69 (52.7)	328 (62.6)		
NRDS	Yes	51 (38.9)	108 (20.6)	19.13	<0.05*
	No	80 (61.1)	416 (79.4)		
Mechanical ventilation	Yes	82 (62.6)	282 (53.8)	3.27	0.07
	No	49 (37.4)	242 (46.2)		

* Statistical significance (*P* < 0.05).

### Laboratory parameters

The mean umbilical artery PH was significantly lower (7.23 ± 0.14) in the brain-injury group compared to the no-brain-injury group (7.26 ± 0.13, *t* = −2.45, *P* = 0.01). Similarly, umbilical artery BE (−4.92 ± 1.63 mmol/L) was significantly lower in the brain-injury group (*t* = −2.08, *P* = 0.03). The median blood glucose level was significantly lower (3.28 mmol/L [IQR: 3.11–3.89]) in the brain-injury group compared with the no-brain-injury group (4.11 mmol/L [IQR: 3.76–4.61], *Z* = −10.11, *P* < 0.001). Additionally, serum concentrations of BDNF and 25-hydroxyvitamin D [25(OH)D] were significantly lower in the brain-injury group (both *P* < 0.05, Table 4).

**Table 4:** Comparison of umbilical artery blood gas and blood routine tests between brain injury and non-brain injury groups in high-risk neonates.

Variables		Brain injury group (*n* = 131)	Non-brain injury group (*n* = 524)	*t*/*z*	*P*
Umbilical artery blood gas	PH	7.23 ± 0.14	7.26 ± 0.13	−2.45	0.01*
	PaO_2_ (mmHg)	75.28 ± 12.08	77.15 ± 11.61	−1.63	0.11
	PaCO_2_ (mmHg)	45.62 ± 6.58	46.23 ± 6.67	−0.94	0.34
	BE (mmol/L)	−4.84 ± 1.63	−4.59 ± 1.51	−1.69	0.09
Blood routine	RBC (10^12^/L)	4.46 ± 0.38	4.48 ± 0.43	−0.28	0.78
	WBC (10^9^/L)	9.20 (8.30, 10.40)	9.30 (6.50, 11.2)	−1.36	0.17
	Hemoglobin (g/L)	164.62 ± 26.51	163.01 ± 14.56	0.93	0.34
	Platelet (10^9^/L)	245.56 ± 37.78	241.77 ± 35.84	1.07	0.28
Umbilical artery biochemistry (other laboratory indicators)	ALT (U/L)	10.86 ± 4.18	11.03 ± 5.54	−033	0.74
	AST (U/L)	56 (34, 83)	43 (36, 82)	−0.27	0.78
	ALB (g/L)	31.29 ± 2.87	30.85 ± 3.36	1.36	0.17
	GLU (mmol/L)	3.28 (3.11, 3.89)	4.11 (376, 4.61)	−10.11	<0.01*
	CRP (mg/L)	1.46 ± 0.44	1.42 ± 0.41	1.21	0.22
	BDNF (ng/mL)	38.53 ± 5.95	44.84 ± 6.40	10.232	<0.01*
	25(OH)D (nmol/L)	49.37 ± 8.20	63.22 ± 9.71	15.038	<0.01*

* Statistical significance (*P* < 0.05).

### Comparison of maternal clinical characteristics

Maternal clinical data were compared between mothers of high-risk infants with and without brain injury. The prevalence of maternal anemia complicating pregnancy was significantly higher in the brain-injury group compared with the no-brain-injury group (*χ*^2^ = 10.76, *P* = 0.01, Table 5).

**Table 5:** Comparison of maternal clinical data between brain injury and non-brain injury groups in high-risk neonates.

Variables		Brain injury group (*n* = 131)	Non-brain injury group (*n* = 524)	*χ* ^2^	*P*
Maternal age	<35	114 (87.0)	467 (89.1)	0.46	0.49
	≥35	17 (13.0)	57 (10.9)		
Antenatal medication history	Yes	21 (16.0)	103 (19.7)	0.89	0.34
	No	110 (84.0)	421 (80.3)		
Pregnancy complicated by hypertension	Yes	31 (23.7)	98 (18.7)	1.64	0.21
	No	100 (76.3)	426 (81.3)		
Pregnancy complicated by diabetes	Yes	8 (6.1)	50 (9.5)	1.53	0.21
	No	123 (93.9)	474 (90.5)		
Pregnancy complicated by anemia	Yes	27 (20.6)	53 (10.1)	10.76	0.01*
	No	104 (79.4)	471 (89.9)		

* Statistical significance (*P* < 0.05).

### Logistic regression analysis of risk factors

As shown in Table 6, MSAF, NRDS, and maternal anemia complicating pregnancy were independent risk factors for brain injury among NICU high-risk infants (all *P* < 0.05). A higher 5-minute Apgar score was protective; higher scores correlated with a lower probability of brain injury (*P* = 0.01). Additionally, Decreased umbilical artery PH and decreased BE were associated with a higher risk of brain injury (both *P* < 0.05). Conversely, elevated serum concentrations of BDNF and 25-hydroxyvitamin D [25(OH)D] were protective factors, with higher levels correlating with lower risks (both *P* < 0.05).

**Table 6:** Logistic regression analysis of risk factors for brain injury among high-risk neonates.

Variables	*B*	SE	Wald	*P*	OR	95% CI
Umbilical cord abnormality	−0.12	0.48	0.07	0.79	0.88	0.35–2.26
MSAF	0.68	0.30	5.14	0.02*	1.97	1.09–3.56
Intrauterine distress	0.45	0.56	0.65	0.42	1.56	0.53–4.64
5-Minute Apgar score	−0.13	0.03	9.89	0.01*	0.88	0.81–0.95
Pulmonary infection	0.16	0.38	0.18	0.67	1.18	0.56–1.46
NRDS	1.03	0.36	8.18	0.01*	2.79	1.38–5.65
PH	1.72	0.84	4.23	0.06	5.57	0.98–28.57
BE	0.15	0.07	4.23	0.04*	1.16	1.01–1.33
GLU	−1.39	0.16	80.07	<0.01*	0.25	0.18–0.34
Pregnancy complicated by anemia	1.08	0.54	4.06	0.04*	2.94	1.03–8.42
BDNF	−0.58	0.20	8.41	<0.01*	0.56	0.38–0.82
25(OH)D	−0.48	0.19	6.37	<0.01*	0.62	0.43–0.89

* Statistical significance (*P* < 0.05).

### Development and validation of a risk-prediction model

#### Model construction

Using partial regression coefficients obtained from logistic regression, the following predictive equation was developed:

Variables were coded as follows: MSAF (yes = 1, no = 0); NRDS (yes = 1, no = 0); maternal anemia (yes = 1, no = 0).

$$P=1/(1+e^{-y})$$

*Y* = logit(*P*) = −4.224 + 0.68·(MSAF)—0.130·(5-min Apgar score) + 1.030·(RDS) + 0.150·(BE, mmol/L)—1.390·(blood glucose, mmol/L) + 1.080·(maternal anemia)—0.580·(BDNF, ng/mL)—0.480·[25(OH)D, ng/mL].

*P* represents the estimated probability of brain injury, increasing monotonically with *Y*, indicating that larger logit (P) values correspond to higher risk.

#### Visualization of the risk-prediction model (nomogram)

**Figure 1:**
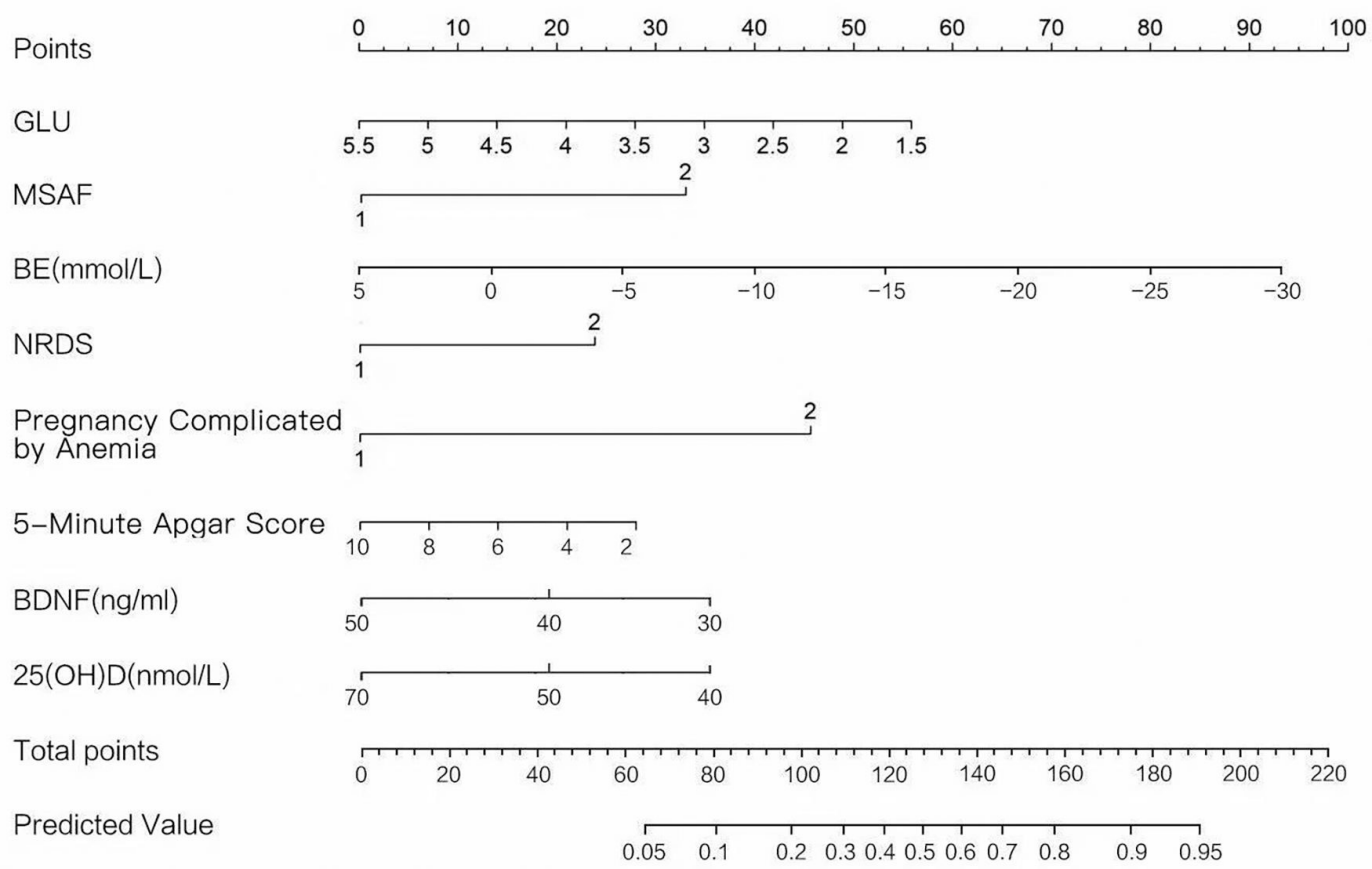
Nomogram for predicting the risk of brain injury in high-risk neonates.

The predictive model was visualized as a nomogram, translating logistic regression outcomes into an intuitive, point-based risk calculator (Figure 1). The upper “Points” axis (0–100) quantifies each perinatal variable's independent contribution. For example, a blood glucose level of 2.5 mmol/L corresponds to 40 points, with larger deviations from normal resulting in higher scores. The middle “Total Points” axis (0–220) sums these individual points, mapping the aggregate directly to the lower “Predicted Probability” axis (0.05–0.95, i.e., 5%–95%).

Illustrative scenario: an infant presenting with RDS (22 points), MSAF (30 points), hypoglycemia (blood glucose 2.5 mmol/L → 40 points), and metabolic acidosis (BE −10 mmol/L → 40 points) would accumulate a total of 132 points, corresponding to an estimated 55% probability of brain injury.

### Evaluation and validation of the risk prediction model

#### Evaluation of the model

**Figure 2:**
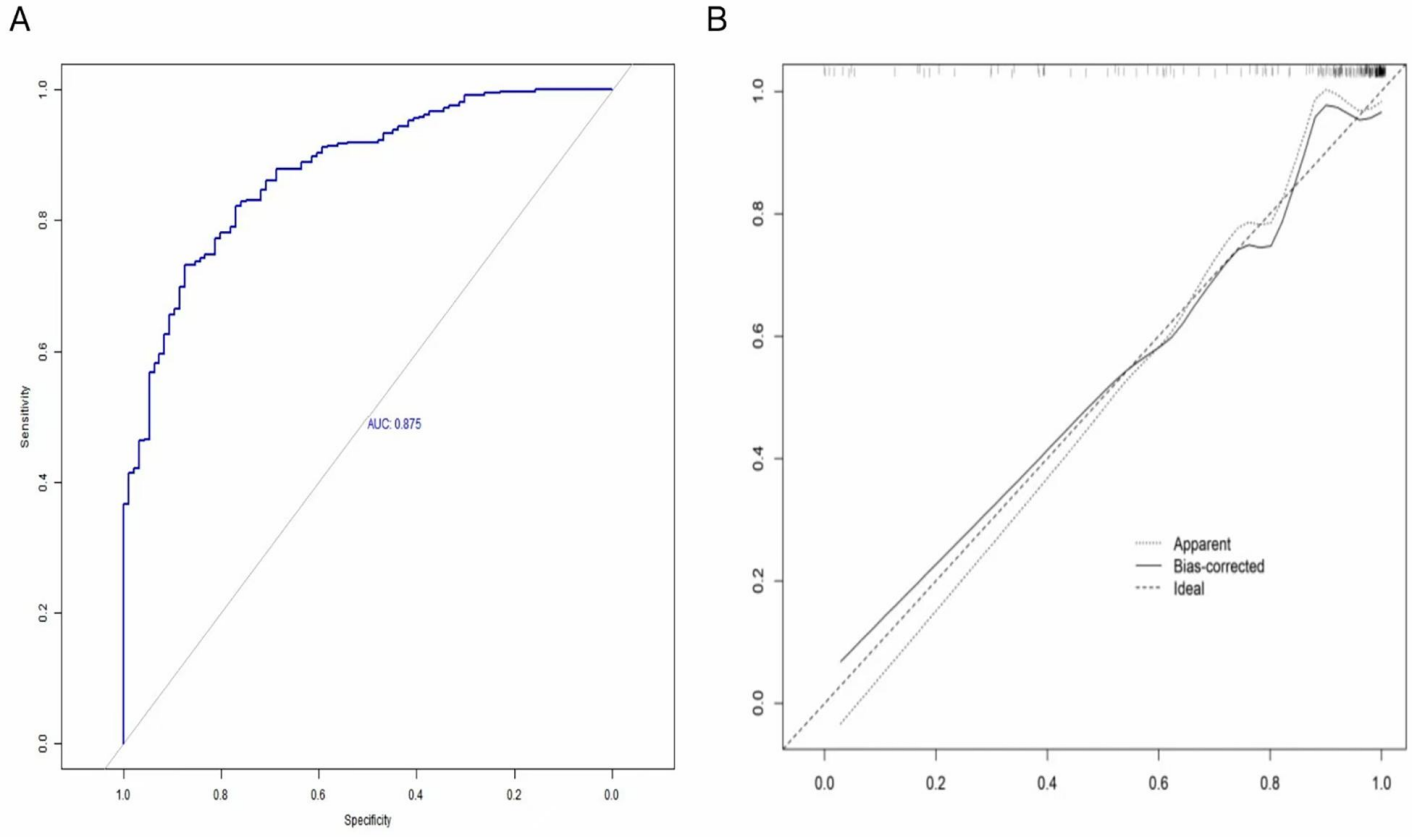
**(A)** ROC curve of the prediction model for brain injury in high-risk neonates; **(B)** calibration curve of the risk-prediction model for brain injury in high-risk neonates.

ROC analysis demonstrated excellent discriminatory performance. The AUC was 0.935, surpassing the threshold of outstanding accuracy (AUC > 0.9). The ROC curve displayed the typical left-upper bulge: at a fixed specificity of 0.80 (20% false-positive rate), sensitivity exceeded 0.90. Thus, the model correctly identified over 90% of true brain-injury cases while maintaining a low false-positive rate. The ROC curve significantly deviated from the reference diagonal (*P* < 0.05), indicating high predictive capability, substantially better than chance. With an AUC of 0.935, the model can effectively limit the false-negative rate to ≤6.5%, strongly supporting its clinical utility (Figure 2A).

The calibration curve was generated through bootstrap resampling (*B* = 500). Predictions closely matched observed event rates across all risk levels, with minimal average prediction error. The Hosmer–Lemeshow goodness-of-fit test (*χ*^2^ = 4.13, df = 8, *P* = 0.85) further confirmed excellent calibration, indicating accurate estimation of neonatal brain-injury risk across the spectrum (Figure 2B).

#### Model validation

**Figure 3:**
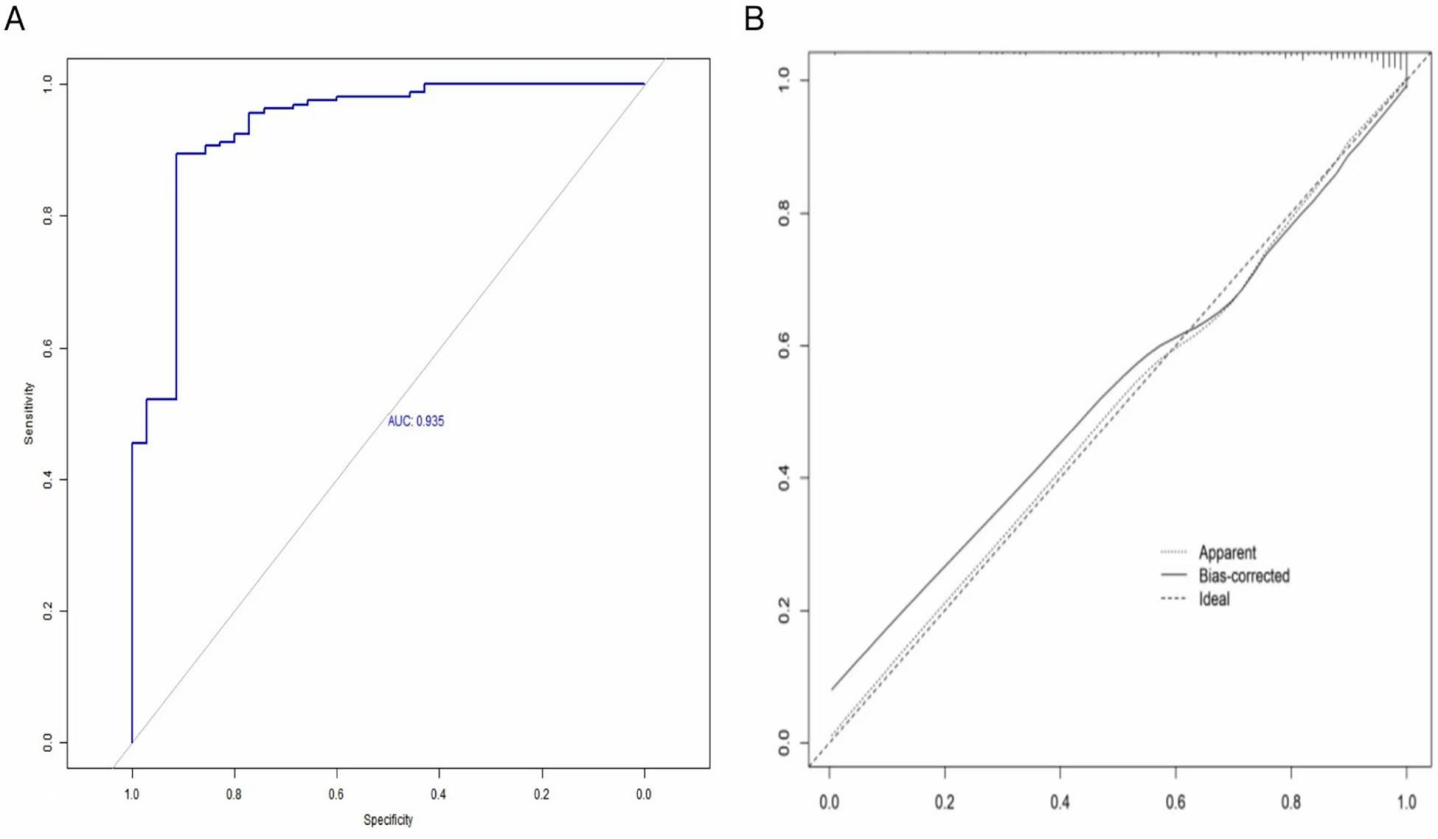
**(A)** ROC curve of the validation cohort for the prediction model; **(B)** calibration curve of the validation cohort for the prediction model.

Internal validation was conducted using a separate cohort of 164 high-risk neonates. ROC analysis in the validation set indicated that the model maintained excellent discriminatory ability (AUC = 0.87). The curve remained substantially above the reference diagonal, with a characteristic upper-left bulge, confirming that predictive performance significantly exceeded random chance. At a specificity of 0.80 (20% false-positive rate), sensitivity reached 0.85, indicating that the model correctly identified 85% of neonates at risk of brain injury. Sensitivity increased to 0.90 when specificity declined to 0.60 (40% false-positive rate) (Figure 3A).

The Hosmer–Lemeshow goodness-of-fit test demonstrated satisfactory calibration (*χ*^2^ = 9.09, df = 8, *P* = 0.34), confirming no significant differences between predicted probabilities and observed risks (*P* > 0.05). This result was further supported by the calibration plot: the ideal calibration line closely matched the observed curve (Apparent). The bias-corrected curve closely overlapped the ideal curve across the entire risk range (0.0–0.4), especially within the high-risk region (>0.2). The largest deviation between the bias-corrected and ideal curves in the moderate-risk range (0.1–0.2) was less than 0.03, and prediction errors in the low-risk range (<0.1) remained below clinically acceptable thresholds (<5%). These findings confirmed the model's clinical utility and strong concordance between predicted and observed risks (Figure 3B).

## Discussion

Neonatal brain injury refers to non-progressive damage occurring from the prenatal period through the first 28 days after birth. It disrupts normal neurodevelopment, often causing delayed psychomotor development and, in severe cases, permanent intellectual disability. Without timely intervention, affected infants are at high risk for lifelong neurological sequelae, including global developmental delay, cognitive impairment, and epilepsy, significantly compromising quality of life.

In this study, 655 high-risk neonates admitted to the NICU were divided into brain-injury and no-brain-injury groups according to clinical diagnosis. Logistic regression analysis identified MSAF, RDS, and maternal anemia complicating pregnancy as independent risk factors for brain injury. Neonatal brain injury has multifactorial pathogenesis. Previous studies also implicated antenatal intrauterine infection, genetic predisposition, intrapartum hypoxia–ischemia, prematurity, and postnatal hyperbilirubinemia in its development.

MSAF refers to the entry of meconium or fetal debris into the amniotic cavity, resulting in contamination. This condition can occur prenatally or intrapartum, commonly after 34 weeks of gestation. Infants exposed to MSAF have an increased risk of aspirating contaminated fluid (20). This study identified MSAF as a predictive factor for brain injury among high-risk neonates in NICUs, consistent with research by Huang Run-zhi et al. However, Zhang et al. (21) reported no association between MSAF and brain injury in preterm infants. Therefore, further research is necessary to clarify the role of MSAF as a causative factor for neonatal brain injury.

The Apgar score, developed by American physician Virginia Apgar (22), is a standardized tool for assessing neonatal status immediately after birth. Initial studies by Woodward et al. (23). indicated a significant correlation between Apgar scores (at 1 and 5 min) and the severity of hypoxic–ischemic encephalopathy (HIE). Additionally, the 5-minute Apgar score correlated with short-term treatment outcomes. Our findings confirm that a higher 5-minute Apgar score corresponds to a lower likelihood of brain injury. However, reliance on the Apgar score alone to diagnose perinatal asphyxia or predict perinatal brain injury has clear clinical limitations. Therefore, combining additional predictive factors with Apgar scores can improve clinical assessments.

NRDS is a severe respiratory condition frequently encountered in neonatal intensive care. Inadequate pulmonary surfactant production, due to immature type II alveolar epithelial cells, causes progressive dyspnea shortly after birth. Severe cases may progress to acute respiratory failure (24). Our findings indicate that NRDS is a significant risk factor for brain injury in high-risk neonates. Infants with NRDS commonly experience asphyxia and hypoxic–ischemic episodes, disrupting cerebral autoregulation. Additionally, stress mediators released due to NRDS may destabilize systemic blood pressure in preterm infants. Both mechanisms increase the risk and severity of ischemic brain injury or intracranial hemorrhage (25).

BE refers to the amount of acid or base (mmol/L) required to titrate a blood sample to PH 7.4 under standard conditions (37 °C–38 °C, PCO_2_ 5.33 kPa/40 mmHg). The normal neonatal range is −10 to −2 mmol/L (26). Our study found that brain injury incidence increased significantly with decreasing BE values, identifying BE as an independent risk factor. This aligns with findings by Lorain et al. (27), suggesting reduced BE as a potential laboratory marker for predicting brain injury in high-risk neonates. Therefore, rapid arterial-blood-gas monitoring is recommended upon NICU admission, enabling timely correction of hypoxemia and acidosis, potentially reducing the incidence of hypoxic–ischemic brain injury.

Although normal neonatal blood glucose levels are 3.9–6.1 mmol/L and hypoglycemia is defined as <2.2 mmol/L (40 mg/dL), our study unexpectedly found significantly lower blood glucose concentrations in the brain-injury group compared to the no-injury group. Thus, close monitoring and rapid correction of abnormal blood glucose levels may be crucial for reducing brain injury incidence. Maternal anemia complicating pregnancy reduces uteroplacental perfusion, decreasing fetal oxygen delivery and impairing fetal growth and development. In this study, maternal anemia prevalence was significantly higher in the brain-injury group compared to the no-brain-injury group, suggesting that infants born to anemic mothers have increased risks of developing brain injury.

BDNF is an essential neurotrophin involved broadly in the growth and differentiation of sympathetic and specific sensory neurons. It plays a crucial role in neuronal outgrowth, maturation, survival, and synaptogenesis during development (28). A study by He Canlin et al. measured serum BDNF in 307 preterm infants using enzyme-linked immunosorbent assay and found that low levels correlated with an increased incidence of brain injury, consistent with our findings. As a major neurotrophin, BDNF promotes neuronal growth and differentiation, maintains neuronal function, and facilitates recovery after brain injury, supporting its therapeutic potential for brain damage recovery (29).

Vitamin D is a fat-soluble secosteroid produced either from dietary cholesterol intake or through conversion of 7-dehydrocholesterol in the skin via ultraviolet radiation (30). Beyond its traditional peripheral role in calcium homeostasis, vitamin D has multiple neurological functions, including neurotrophic signaling and immune and neuroprotective activities (31, 32). In this study, serum 25-hydroxyvitamin D [25(OH)D] levels were significantly lower in high-risk infants who developed brain injury compared to those without brain injury. These results suggest that vitamin D deficiency is a modifiable risk factor in NICU infants. However, it remains uncertain whether vitamin D supplementation can treat established neonatal brain injury. Limited evidence from animal studies suggests potential benefits of vitamin D on the immature central nervous system. Therefore, multicenter randomized trials are needed to confirm if addressing low vitamin D levels can prevent or mitigate long-term neurological outcomes associated with neonatal brain injury.

The key benefit of the nomogram developed in this study is its transformation of a complex logistic regression equation into an intuitive, visual instrument. This significantly enhances readability. Clinicians can quickly estimate an individual infant's probability of brain injury without calculations, facilitating personalized risk assessment and routine clinical application. The model underwent comprehensive evaluation and validation. Both discrimination (the ability to differentiate between infants who will or will not develop brain injury) and calibration (agreement between predicted probabilities and observed outcomes) were satisfactory. These findings confirm that the model can accurately predict the risk of brain injury, providing clinicians with an evidence-based tool for clinical decision-making. Additionally, the model has practical advantages. All predictors involve easily accessible historical data collected routinely during prenatal care or typical laboratory tests, such as complete blood counts and blood gas analyses, performed regularly in clinical practice. No specialized tests are required, making data collection straightforward and cost-effective. Clinicians can easily input routinely collected infant data into the nomogram to promptly determine brain injury risk. These risk classifications (high, moderate, or low) inform clinical decisions for early intervention, significantly improving care effectiveness.

Despite these findings, several limitations of the study exist. First, the study was retrospective and conducted at a single center, thus evaluating only relationships between clinical and laboratory variables and brain injury without considering other potential influences, such as prenatal nutrition and genetic factors. Second, since participants were recruited from a single institution, findings are susceptible to selection bias and may lack generalizability. Third, the analysis treated brain injury as a single outcome, although risk factors differ among hemorrhagic, ischemic, and other subtypes. Subtype-specific analyses were not performed; thus, results cannot guide targeted preventive or management strategies. In conclusion, by analyzing clinical records and laboratory data from 655 high-risk NICU infants, this study identified factors influencing neonatal brain injury and provided evidence aiding clinicians in early identification. Future multicenter, large-scale studies are required to ensure timely diagnosis, detection, and treatment of neonatal brain injury.

## Data Availability

The datasets presented in this study can be found in online repositories. The names of the repository/repositories and accession number(s) can be found in the article/Supplementary Material.
